# Comparable prevalence of distant metastasis and survival of different primary site for LN + pancreatic tumor

**DOI:** 10.1186/s12967-020-02438-1

**Published:** 2020-07-01

**Authors:** Xin Lou, Jun Li, Ya-Qing Wei, Zhi-Jia Jiang, Ming Chen, Jin-Jin Sun

**Affiliations:** Department of Hepatopancreatobiliary Surgery, The Second Hospital of Tianjin Medical University, Tianjin, China

**Keywords:** SEER, Pancreatic cancer, Prevalence, Mortality, Epidemiology

## Abstract

**Background:**

Few studies have delved into the prevalence of distant metastasis (DM +) and survival for patients with lymph node metastases (LN +) by primary site. We aimed to detect differences in distant metastasis and prognosis between pancreatic head and bodytail tumors for LN + patients.

**Methods:**

Patients with chemotherapy, histologically diagnosed, primary site between 2004 and 2016 were included from the SEER (Surveillance, Epidemiology, and End Results) database. Pancreatic head tumors were compared with pancreatic bodytail tumors using the odds ratio (OR) for rates of distant metastasis, hazard ratios (HR) for overall survival (OS) and cancer-specific survival (CSS). The competing risk model and propensity score matching (PSM) were performed to further explore.

**Results:**

Of 5726 LN + patients identified from the SEER database, pancreatic head tumors account for 85.2% (4877 of 5726) and 14.8% (849 of 5726) were pancreatic bodytail tumors. The incidence of DM was lower in pancreatic head than in pancreatic bodytail tumors (OR, 0.29; 95% CI 0.23–0.37; *P* < 0.001). The multivariate Cox regression show pancreatic head tumors have a significantly shorter survival rate relative to pancreatic bodytail (HR, 1.12; 95% CI 1.03–1.22; *P* = 0.008), but the primary site was not a significant independent risk factor for prognosis by log-rank test (*P* = 0.39) and multivariate competing risk model [subdistribution HR (SHR), 1.08; 95% CI 0.98–1.19; *P* = 0.087].We then examined our conclusion by 1:1 propensity score matching, and the result reflected pancreatic head tumors have a lower risk of DM compared with pancreatic bodytail tumors (OR, 0.22; 95% CI 0.15–0.34; *P* < 0.001), but the primary site of pancreatic tumors was not associated with LN + patient survival based on univariate Cox regression (HR, 1.04; 95% CI 0.93–1.17; *P* = 0.435) and competing risk analysis (SHR, 1.01; 95% CI 0.89–1.12; *P* = 0.947).

**Conclusions:**

LN + pancreatic head tumors were significantly lower risk of DM relative to pancreatic bodytail tumors. Survival outcome in LN + pancreatic tumors didn’t exist significant differences split by primary site, which indicates that the prognosis of LN + patients with chemotherapy isn’t associated with the primary site of metastasis, but with the occurrence of metastasis.

## Background

Pancreatic tumor was considered to generate from well-defined precursor, and it needs many years to develop malignant tumors from non-invasive precursor [1]. However, the progression from early to middle even advanced stage was very fast. In most cases, because pancreatic tumor is difficult to detect and the early symptoms are atypical, local progression or metastasis have occurred at the time of diagnosis and more than 80% of patients aren’t amenable to surgical resection [2, 3]. Though rate of early diagnosis improving was urgent, it is also very important to better understand the occurrence for distant metastases and promote the correct treatment strategy for patients in a timely manner. Tumor size was an independent risk factor for the occurrence of distant metastasis in pancreatic cancer, and the size of less than 5 cm was higher rate of distant metastasis compared with other size [4]. Previous study also has reported that the younger age, sex, larger size, low ALT and high CA19-9 are thought to be associated with distant metastasis [5]. Unluckily, so far very few studies have explored the risk factors of distant metastasis for LN + pancreatic tumors. Factors related to distant metastasis of pancreatic cancer have not been conclusively determined.

Lots of studies have indicated that right and left colon tumors have different clinical survival features [6–8], implying that carcinogenesis may occur variation in different primary locations of the same tumor. The pancreas was composed of multiple anatomic regions including head, body and tail. Whether the primary site has an impact on the prognosis of pancreatic tumors remains controversial. The 3 year survival rate for pancreatic bodytail is higher compared with that pancreatic body tumors in local stage (20 versus 9%), while the survival rate of pancreatic body was higher than that of pancreatic head tumor with regional (6.7 versus 7.6%) or distant metastasis (1.4 versus 1.8%) [9]. Another study reported the survival of pancreatic body tumor was higher survival rate compared with pancreatic head tumor in stage I, and lower in stage II–IV, as for pancreatic tail tumor, the survival rate was higher than pancreatic head tumor in all stages [10]. However, Birnbaum and his colleague thought no matter which stages the pancreatic tumor is in, the overall survival was better for the head than for bodytail tumors [11]. In contrast, other studies present that survival rate between head and bodytail tumors was similar [12, 13]. These studies focusing on the association between the primary site and clinical outcome are extremely controversial [14, 15].

In this study the patients from the SEER database were used to analyze whether the primary site was associated with DM rate and survival rate. The risk factors for distant metastasis and survival rate were also explored based on multivariate regression. Our conclusion was examined by propensity matching score.

## Methods

### Patients and methods

Anonymized patient-level data were obtained from Surveillance, Epidemiology, and End Results (SEER) database by the National Cancer Institute’s SEER*Stat software based on a private ID. We screened patients aged 18 or more with lymph node metastasis (LN +) diagnosed between 2004 and 2016. The SEER program collected data on the incidence and survival from US regions accounting for 28% of national population [16]. We enrolled the samples of primary pancreatic tumor with histology diagnosis or microscopically confirmed diagnosis. Tumor stage was coded according to the American Joint Committee on Cancer (AJCC) TNM staging system, 7th edition [17]. Patients with cancer diagnosed on autopsy or death certificate were excluded and we also excluded patients with Stage I and Stage IIA. Patients without chemotherapy were excluded from our study.

### Statistical analysis

The primary endpoint was the distance metastasis (DM), overall survival (OS) and cause-specific survival (CSS). The main explanatory variable was survival after chemotherapy, split by the primary site (Head and Bodytail). The differences between demographic groups were tested by using the Chi-square test. Multivariate logistic regression was conducted to analyze potential risk factors for DM and obtain the odds ratio (OR) with 95% confidence interval (CI). Kaplan–Meier survival curves were used to calculate the 1 year and 3 year OS and CSS rate, and the difference between pancreatic head and bodytail was examined by the log-rank test. The association between all variables and survival rate for LN + patients was evaluated using Cox proportional regression model, with results reported by hazard ratios (HRs) and 95% confidence intervals (CIs). When we considered the death not related to pancreatic tumors, the competing risk regression model was considered as a useful tool to Cox regression which could avoid unbiased hazarded ratio. Then, we explored the risk factors for survival rate using competing risk regression, with results reported by subdistribution hazard ratio (SHRs) and 95% confidence intervals (CIs).

To avoid selecting bias, propensity-score matching (PSM) was performed to compare distant metastasis and survival rate between pancreatic head and bodytail tumors. Matching was based on propensity scores obtained by logistic regression model and using one-to-one nearest neighbor matching as the dependent variable. Match tolerance of 0.01 was used as the cut-off value and satisfactory matching was obtained under circumstance of the standardized difference in means ≤ 0.1. We used univariate logistic regression and Chi-square test to explore the relationship of the primary site with the status of DM based on selected samples after PSM. For the survival rate between pancreatic head and bodytail tumor, comparison of two subgroups was performed with the log-rank test, univariate Cox regression, and visualized with Kaplan–Meier plots.

All analyses were performed with IBM Stata (Version 16.0, StataCorp LLC), R statistical software (version 3.5.2, StataCorp LLC, College Station,Tex), and propensity score matching was conducted with SPSS (version 22.0, IBM Corp, Armonk, NY), with 2-sided p value less than 0.05 considered statistically significant.

## Results

### Patient demographic and tumor characteristics

**Fig. 1:**
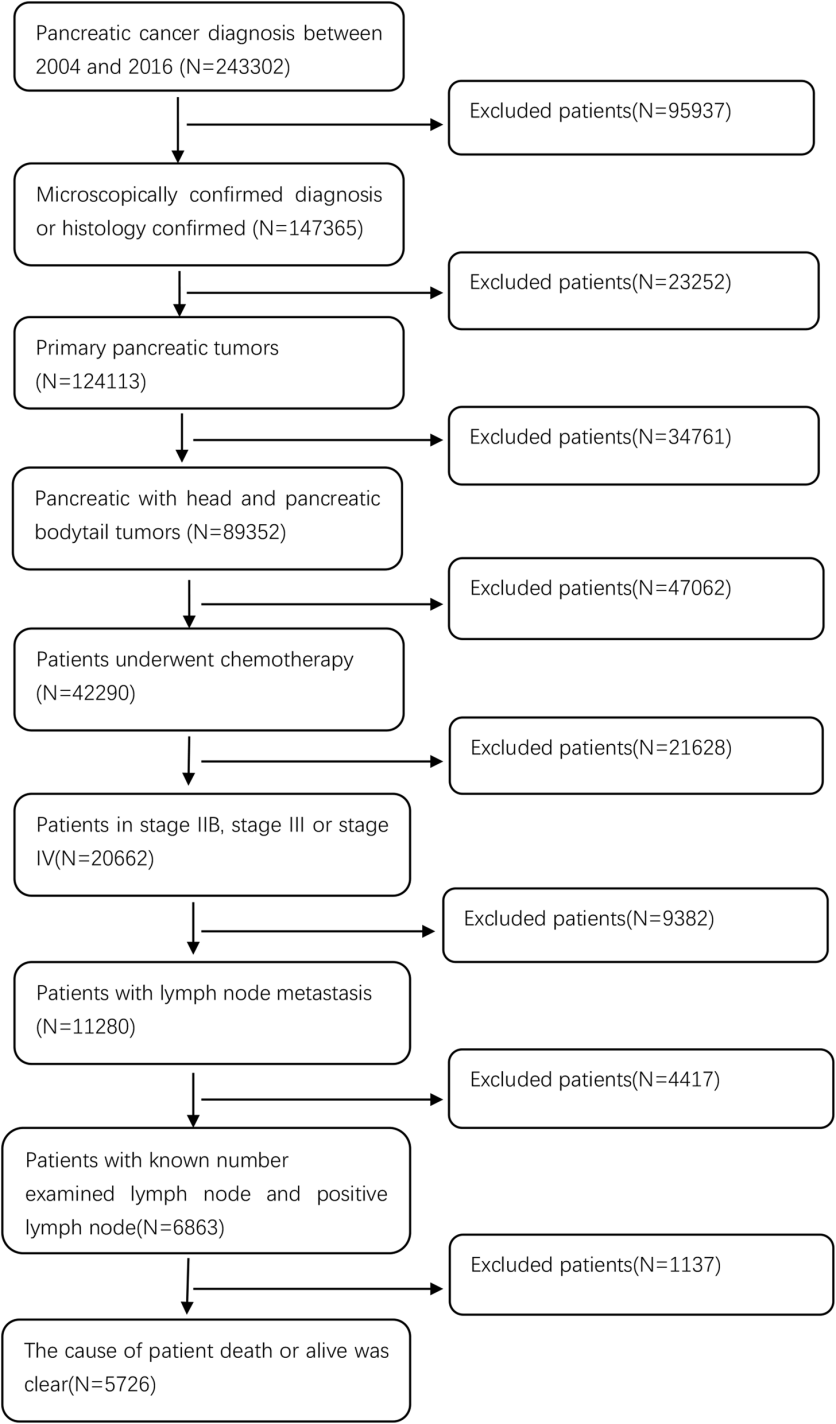
Flowchart of selection of LN + patients with pancreatic tumors

We obtained 5726 samples who comply with the screening criteria (Fig. 1), in which there were 409 (7.1%) patients with distant metastasis (DM +) and 5317 (92.9%) with free distant metastasis. 2–4 cm tumors were most common in patients with lymph node positivity (3498 of 5726, 61.1%), compared with ≤ 2 cm tumors (716 of 5726, 12.5%) and > 4 cm tumors (1512 of 5726, 26.4%). The lymph node ratio was at an increasing trend in DM + patients. 17.7% (134 of 755) patients with > 50% lymph node ratio was DM + patients, while 7.2% (204 of 3498) patients with 25–50% lymph node ratio was DM + patients and 4.7% patients (166 of 3466) with < 25% lymph node ratio was DM + patients. More detail can be obtained from Table 1. We split the LN + patients by the primary site to explore the DM + difference between pancreatic head and bodytail tumors (Table 2), LN + patients with pancreatic bodytail tumors occur distant metastasis with a higher probability compared with pancreatic head tumors (17.1 versus 5.4%). We found 2–4 cm pancreatic head tumors are more common (3135 of 4877, 64.3%), while more common size of pancreatic bodytail tumors was larger than 4 cm (432 of 849, 50.9%). Men have a higher frequency of pancreatic head tumors (2563 of 4877, 52.6%), and women developed pancreatic bodytail tumor with a higher probability (439 of 849, 51.7%). Furthermore, median follow-up of entire 24.8 months with range from 1 to 155 for all pancreatic tumors. Of these, the pancreatic bodytail was 24.9 months with range from 1 to 154, and head was 24.7 months with range from 1 to 155.

**Table 1:** Patients clinical features of LN + pancreatic tumors and distant metastasis

	Total	No distance metastasis	Distance metastasis	*P* value
Age				< 0.001
18–49	521 (9.1)	464 (8.7)	57 (13.9)	
50–59	1412 (24.7)	1289 (24.2)	123 (30.1)	
60–69	2074 (36.2)	1948 (36.6)	126 (30.8)	
≥ 70	1719 (30.0)	1616 (30.4)	103 (25.2)	
Sex, no. (%)				0.730
Male	2973 (51.9)	2764 (52.0)	209 (51.1)	
Female	2753 (48.1)	2553 (48.0)	200 (48.9)	
Race, no. (%)				0.384
White	4710 (82.3)	4377 (82.3)	333 (81.4)	
Black	592 (10.3)	553 (10.4)	39 (9.5)	
Other	424 (7.4)	387 (7.3)	37 (9.1)	
Marital status, no. (%)				0.090
Never married	709 (12.4)	657 (12.4)	52 (12.7)	
Married	3868 (67.6)	3576 (67.3)	292 (71.4)	
Divorced/widowed	1149 (20.1)	1084 (20.4)	65 (15.9)	
Size, no. (%)				< 0.001
≤ 2 cm	716 (12.5)	689 (13.0)	27 (6.6)	
≤ 4 cm	3498 (61.1)	3294 (62.0)	204 (49.9)	
> 4 cm	1512 (26.4)	1334 (25.0)	178 (43.5)	
Differentiation, no. (%)				< 0.001
Well	477 (8.3)	436 (8.2)	41 (10.0)	
Intermediate	2590 (45.2)	2464 (46.3)	126 (30.8)	
Poor/undifferentiated	2133 (37.3)	1994 (37.5)	139 (34.0)	
Missing	526 (9.2)	423 (8.0)	103 (25.2)	
Lymph node ratio, no. (%)				< 0.001
1–25%	3466 (60.5)	3300 (62.1)	166 (40.5)	
26–50%	1505 (26.3)	1396 (26.3)	109 (26.7)	
> 50%	755 (13.2)	621 (11.7)	134 (32.8)	
Number of malignant mass, no. (%)				0.047
1	5493 (95.9)	5093 (95.8)	400 (97.8)	
> 1	233 (4.1)	224 (4.2)	9 (2.2)	
Primary site no. (%)				< 0.001
Pancreas head	4877 (85.2)	4613 (86.8)	264 (64.5)	
Pancreas body tail	849 (14.8)	704 (13.2)	145 (35.5)	
Stage, no. (%)				< 0.001
IIB	5003 (87.3)	5003 (94.1)	0	
III	314 (5.5)	314 (5.9)	0	
IV	409 (7.2)	0	409 (100%)

**Table 2:** Patients clinical features of LN + pancreatic tumors split by primary site

	Total	Pancreas head	Pancreas body tail	*P* value
Age group, no. (%)				0.293
18–49	521 (9.1)	434 (8.9)	87 (10.2)	
50–59	1412 (24.7)	1215 (24.9)	197 (23.2)	
60–69	2074 (36.2)	1778 (36.5)	296 (34.9)	
≥ 70	1719 (30.0)	1450 (29.7)	269 (31.7)	
Sex, no. (%)				0.022
Male	2973 (51.9)	2563 (52.6)	410 (48.3)	
Female	2753 (48.1)	2314 (47.4)	439 (51.7)	
Race, no. (%)				0.104
White	4710 (82.3)	4032 (82.7)	679 (80.0)	
Black	592 (10.3)	488 (10.0)	104 (12.2)	
Other	424 (7.4)	357 (7.3)	67 (7.9)	
Marital status, no. (%)				0.310
Never married	709 (12.4)	603 (12.4)	106 (12.5)	
Married	3868 (67.6)	3279 (67.2)	589 (69.4)	
Divorced/ widowed	1149 (20.1)	995 (20.4)	154 (18.1)	
Size, no. (%)				< 0.001
≤ 2 cm	716 (12.5)	662 (13.6)	54 (6.4)	
≤ 4 cm	3498 (61.1)	3135 (64.3)	363 (42.8)	
> 4 cm	1512 (26.4)	1080 (22.1)	432 (50.9)	
Differentiation, no. (%)				0.551
Well	477 (8.3)	399 (8.2)	78 (9.2)	
Intermediate	2590 (45.2)	2197 (45.0)	393 (46.3)	
Poor/undifferentiated	2133 (37.3)	1833 (37.6)	300 (35.3)	
Missing	526(9.2)	448(9.2)	78(9.2)	
Lymph node ratio, no. (%)				
1–25%	3466 (60.5)	2923 (59.9)	543 (64.0)	0.019
26–50%	1505 (26.3)	1315 (27.0)	190 (22.4)	
> 50%	755 (13.2)	639 (13.1)	116 (13.7)	
Number of malignant mass, no. (%)				0.305
One	5493 (95.9)	4684 (96.0)	809 (95.3)	
More than one	233 (4.1)	193 (4.0)	40 (4.7)	
Distance metastasis, no. (%)				< 0.001
No	5317 (92.9)	4613 (94.6)	704 (82.9)	
Yes	409 (7.1)	264 (5.4)	145 (17.1)	
Stage, no. (%)				< 0.001
IIB	4917 (85.8)	4277 (87.7)	640 (75.5)	
III	302 (5.3)	258 (5.3)	44 (5.2)	
IV	507 (8.9)	342 (7.0)	165 (19.3)	

### Analysis of risk factors for distant metastasis of pancreatic tumors

**Fig. 2:**
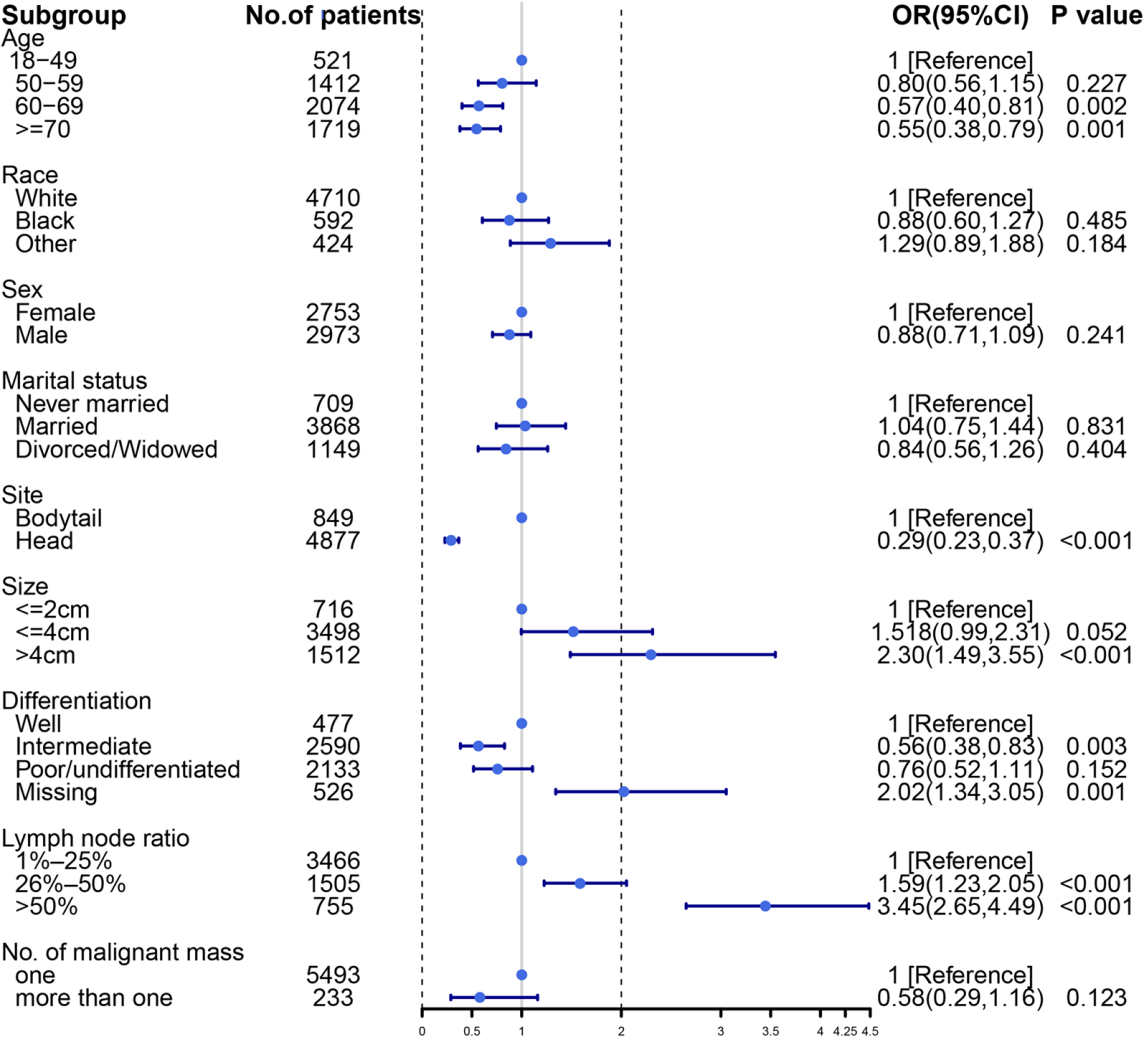
Forest plot to visualize odds ratio of risk factors for distance metastasis for 5726 LN + patients

**Fig. 3:**
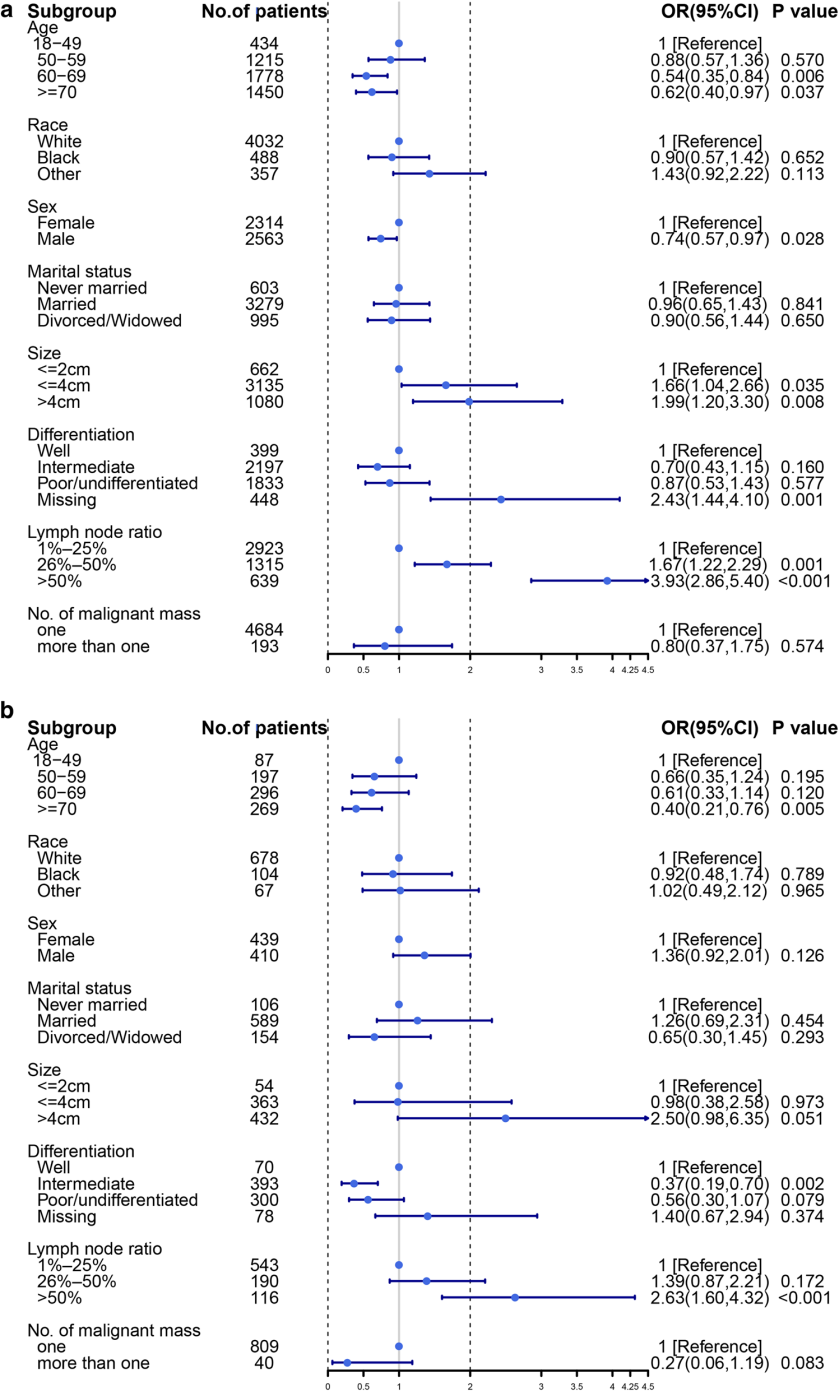
Forest plot to visualize odds ratio of factors for distance metastasis for 4877 LN + patients with pancreatic head tumors (**a**) and 849 LN + patients with pancreatic bodytail tumors (**b**)

We explore risk factors for distant metastasis in LN + patients with pancreatic tumors by a multivariate logistic regression. The incidence of DM was lower in pancreatic head than in pancreatic bodytail tumors (OR, 0.29; 95% CI 22.8–36.8%; *P *< 0.001). For LN + patients with poorly differentiated levels, distant metastasis does not differ between pancreatic head cancer and pancreatic bodytail tumors (OR, 0.75, 95% CI 51.6–110%; *P* = 0.153). More details could be found from Fig. 2. To further identify whether there are differences in risk factors in different primary site, we split the patients into two subgroups and then perform the multivariate logistic analysis separately. Age over 60 years is a risk factor of distant metastasis for LN + pancreatic head tumors, but for pancreatic bodytail tumors, the incidence of distant metastasis was found to not be a risk factor to patients aged 60–70 (*P* = 0.120). Interestingly, only pancreatic bodytail tumor with moderately differentiated cell type was associated with DM, and the degree of tumor differentiation doesn’t have any effect on DM of LN + pancreatic head tumors. More detail could be found from Fig. 3a, b.

**Fig. 4:**
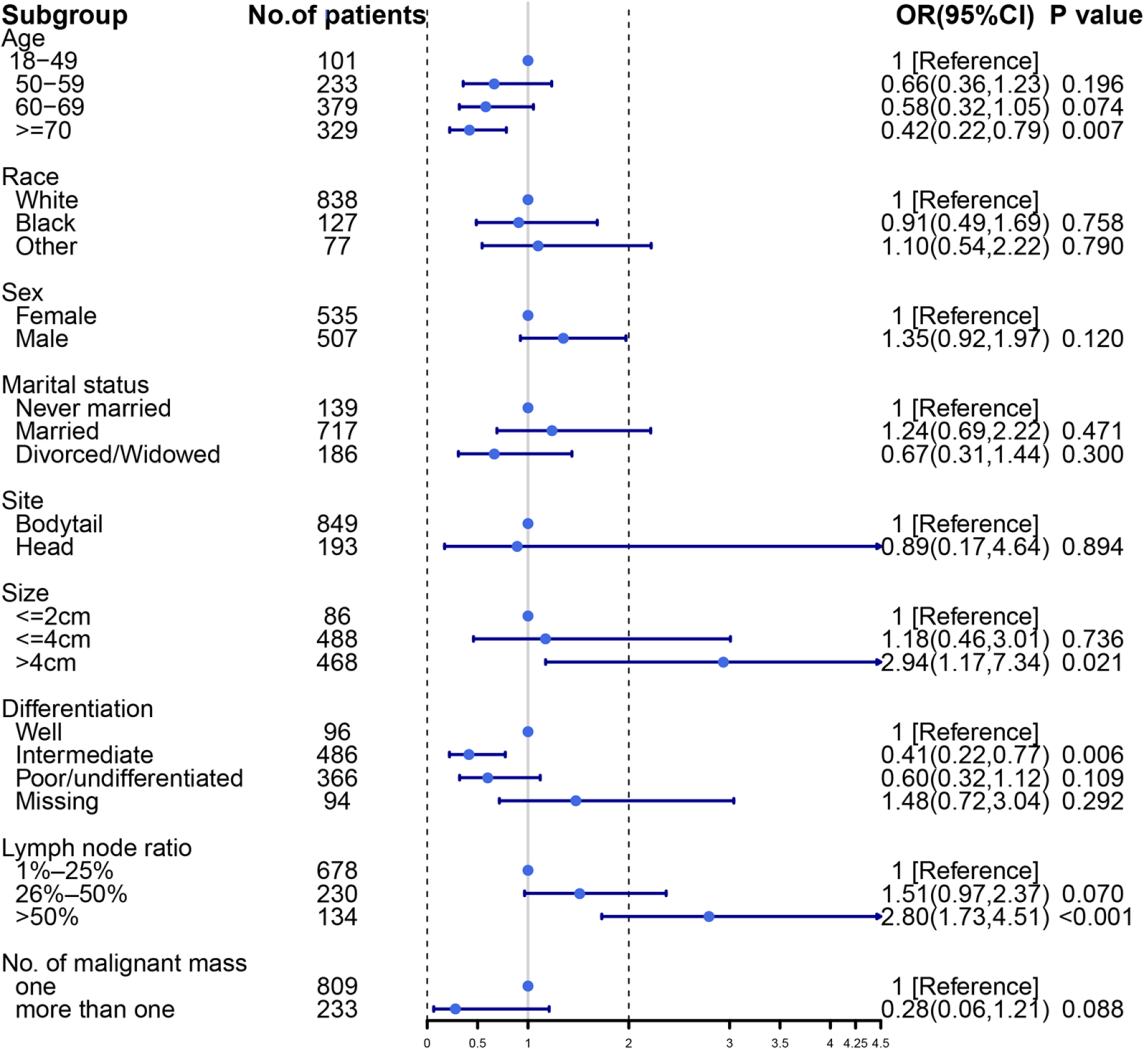
Forest plot to visualize odds ratio of factors for distance metastasis for 1042 LN + patients with pancreatic bodytail and more than one mass head tumors

We found the DM + incidence of pancreatic head tumors was significantly different from that of pancreatic bodytail tumors from Fig. 2. However, while we compared the DM incidence of pancreatic bodytail tumors with that of pancreatic head with more than one malignant mass, the results show that primary site had no relevance to the DM incidence (OR = 0.894, 95% CI 17.2–463.7%; *P* = 0.894). Furthermore, older age, larger size, and higher lymph node ratio were proved to be independent risk factors once again based on subgroup analysis (Fig. 4).

### Survival analysis of LN + pancreatic tumors based on different primary site

**Fig. 5:**
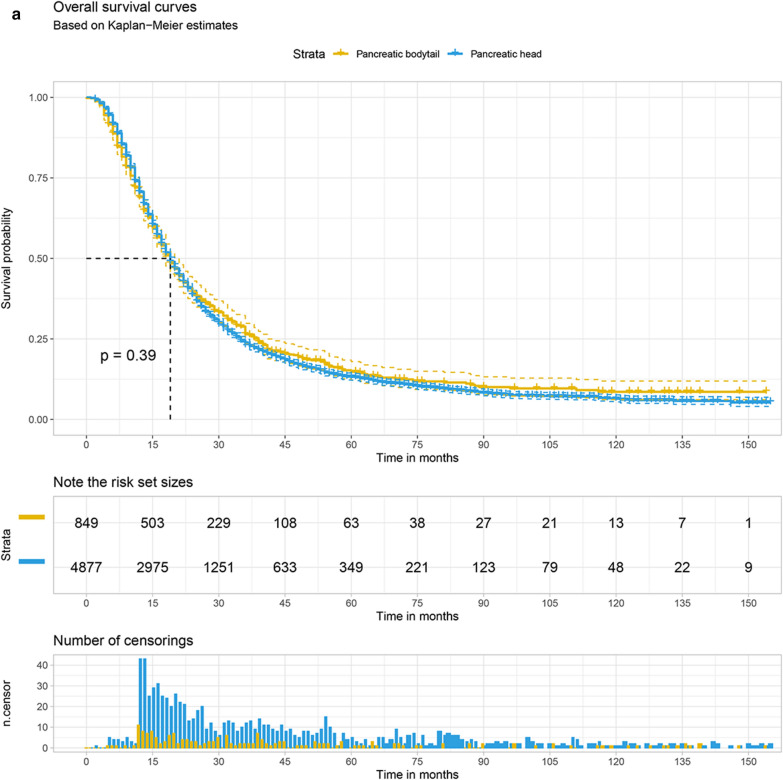

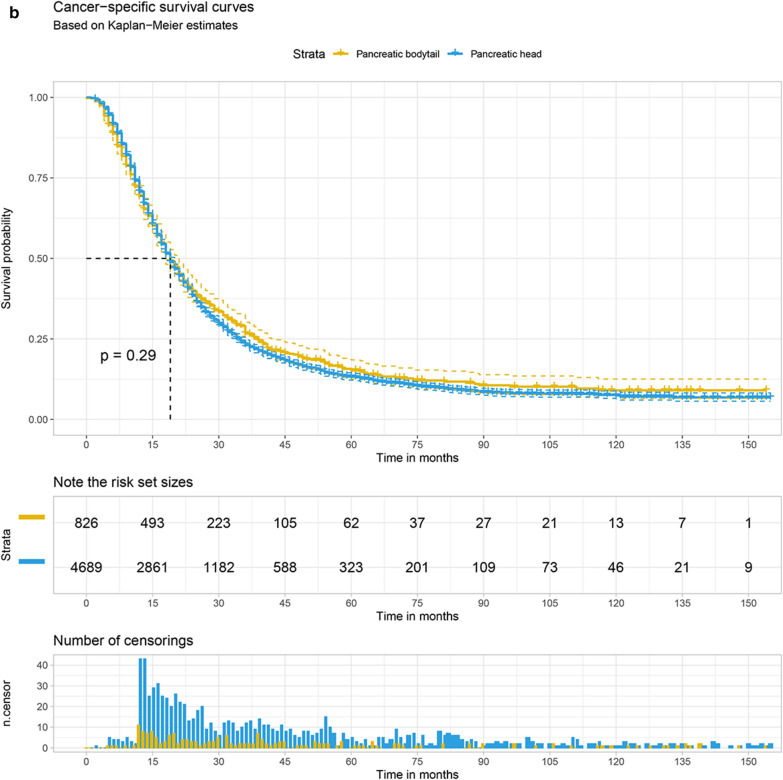
The 1 year and 3 year overall survival (**a**) and cancer-specific survival (**b**) of patients with LN + pancreatic tumors split by primary site

The Kaplan–Meier survival curve was plotted to compare the overall survival (OS) and cancer-specific survival (CSS) of the pancreatic head with that of pancreatic bodytail tumors. The results demonstrate that LN + patients with pancreatic head tumors had the 1 year and 3 year OS at 70.6% (95% CI 69.3–71.8%) and 23.5% (95% CI 22.3–24.8%), respectively. For patients with pancreatic bodytail tumors, the 1 year and 3 year OS were 69.1% (95% CI ,65.8–72.1%) and 26.4% (95% CI 23.3–29.7%). Similar results also were found while we explore the CSS of two different sites pancreatic tumors. Interestingly, the results of log-rank test indicated there was no significant differences in the OS and CSS of two primary sites of pancreatic tumors. More details are available from the Fig. 5a, b.

**Fig. 6:**
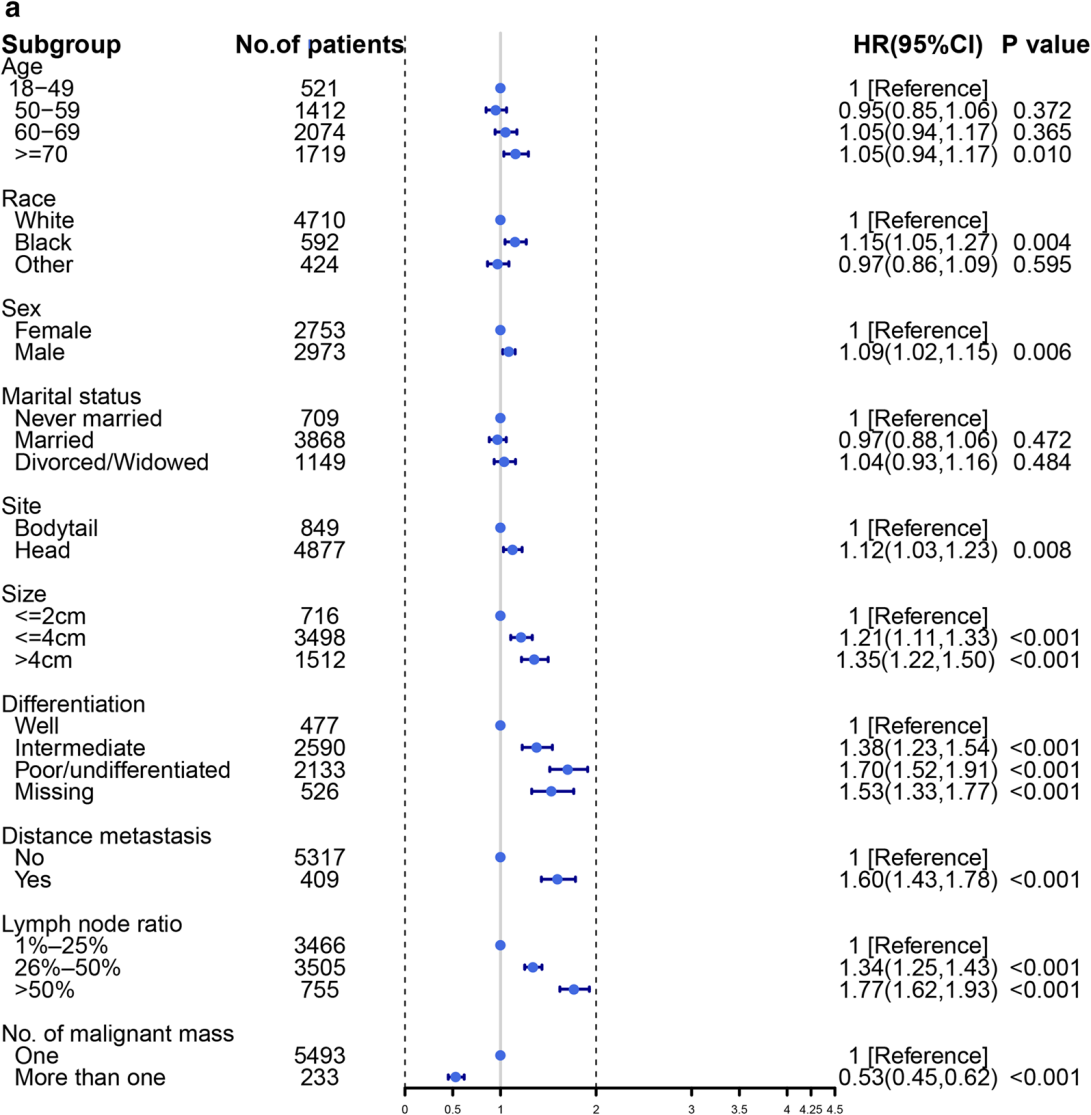

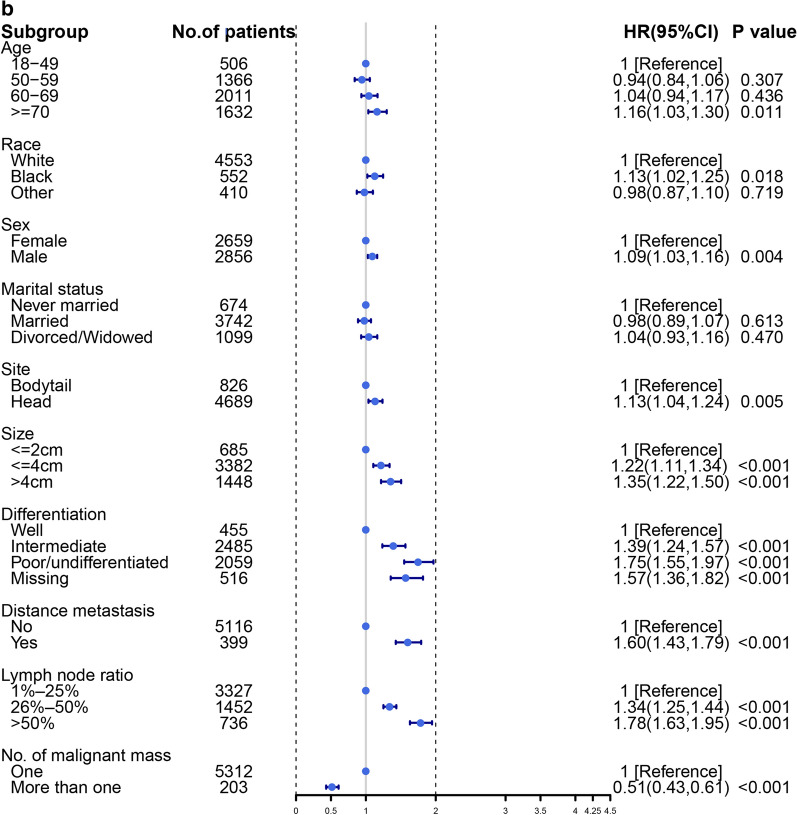
Forest plot to visualize the HR of risk factors for overall survival (**a**) and cancer-specific survival (**b**) of LN + pancreatic tumors

A multivariable Cox regression analysis was performed to explore the risk factors for OS. The results showed that the primary site was a significant independent factor (HR, 1.12; 95% CI 1.03–1.22; *P* = 0.008), which shows the opposite result. The DM of pancreatic tumors was a very strong risk factor associated with the prognosis of patients (HR, 1.59; 95% CI 1.42–1.78; *P *< 0.001). Older age was also a risk factor (HR, 1.15; 95% CI 1.03–1.29; *P* = 0.010). As for race, the black people have more significant shorter OS compared with that of white people (HR, 1.15; 95% CI 1.04–1.26; *P* = 0.004), while other races show the similar OS compared with white people (HR, 0.96; 95% CI 0.86–1.08; *P* = 0.595). More details are available from the Fig. 6a. While we explore the risk factor to CSS, the similar results were found from Fig. 6b.

Considering bias due to death not related to pancreatic tumors, we performed a multivariate competing risk regression to explore the risk factors of survival rate. The results reflected an interesting thing that the pancreatic head tumors have a similar survival rate compared with that of pancreatic bodytail tumors (SHR = 1.08; 95% CI 0.98–1.18; *P* = 0.087). Age isn’t the risk factor for LN + patients. The gender (*p* = 0.047), tumor size (< 0.001), grade (< 0.001), distance metastasis (*p* < 0.001), LNR (*p* < 0.001) and the number of malignant mass (*p* < 0.001) were the dependent risk factors. More details are available from the Table 3.

**Table3:** Predictors of survival rate in LN + pancreatic tumors by competing risk regression

	SHR	95% CI	*P* value
Age, years			
18–49	1 [Reference]		
50–59	0.93	0.83–1.04	0.210
60–69	1.03	0.92–1.14	0.646
≥ 70	1.07	0.96–1.19	0.244
Sex			
Female	1 [Reference]		
Male	1.06	1.01–1.13	0.047
Race			
White	1 [Reference]		
Black	1.04	0.93–1.15	0.510
Other	0.97	0.87–1.09	0.642
Marital status			
Never married	1 [Reference]		
Married	1.01	0.91–1.11	0.881
Divorced/ widowed	1.05	0.94–1.18	0.368
Size, no. (%)			
≤ 2 cm	1 [Reference]		
≤ 4 cm	1.21	1.10–1.32	< 0.001
> 4 cm	1.29	1.16–1.43	< 0.001
Differentiation			
Well	1 [Reference]		
Intermediate	1.36	1.22–1.52	< 0.001
Poor/undifferentiated	1.71	1.53–1.91	< 0.001
Missing	1.58	1.37–1.82	< 0.001
Lymph node ratio			
1–25%	1 [Reference]		
26–50%	1.32	1.24–1.42	< 0.001
> 50%	1.76	1.61–1.93	< 0.001
Number of malignant mass			
One	1 [Reference]		
More than one	0.46	0.39–0.54	< 0.001
Distance metastasis			
No	1 [Reference]		
Yes	1.56	1.37–1.78	< 0.001
Site			
Pancreatic bodytail	1 [Reference]		
Pancreatic head	1.08	0.98–1.187	0.087

### Pancreatic head tumors versus pancreatic bodytail tumors after propensity score matching

**Fig. 7:**
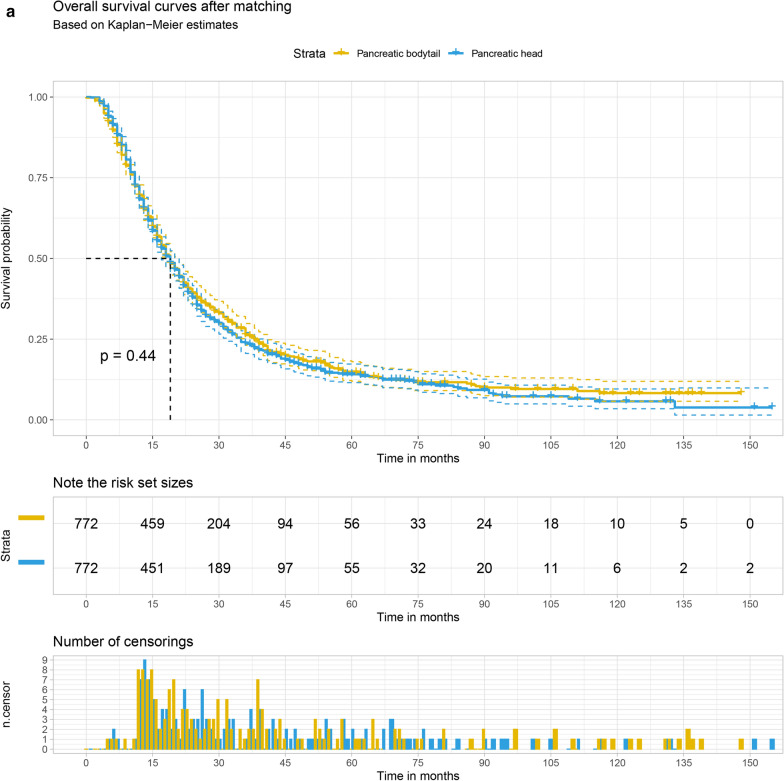

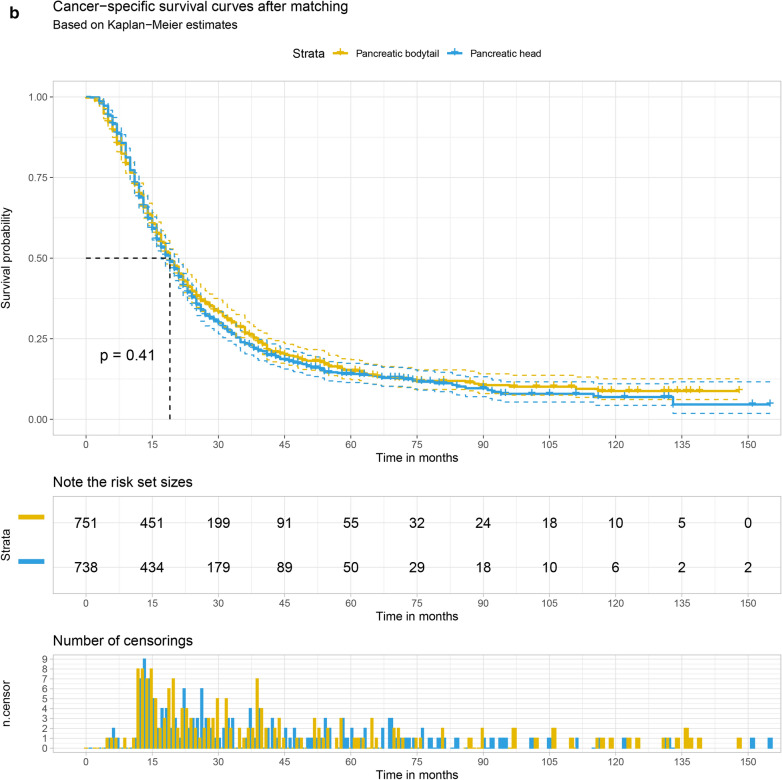
The overall survival (**a**) and cancer-specific survival (**b**) of patients with LN + pancreatic tumors split by primary site after matching

1544 patients were obtained after 1:1 matching, including 772 pancreatic head tumors and 772 pancreatic bodytail tumors. The relative multivariate imbalance L1 statistic value after matching is 0.231, far less than 0.515 before matching, indicating a good match. As shown in Table 4, distribution of variates was adequately balanced in matched data set. Logistic regression analysis was conducted to explore the correlation between the primary site of LN + pancreatic tumors and the occurrence of distant metastases. Pancreatic head tumors have a lower risk of DM compared with pancreatic bodytail tumors (OR, 0.22; 95% CI 0.15–0.34; *P* < 0.001). A significant difference in the incidence of DM was also found between the pancreatic head tumors and pancreatic bodytail tumors by Chi-square test (*P* < 0.001). Univariate Cox regression analysis was performed to explore the correlation between the survival rate and the primary site of LN + pancreatic tumors. There was also no difference in survival rate between pancreatic head tumors and pancreatic bodytail tumors (HR, 1.04; 95% CI 0.93–1.17; *P* = 0.435). Similar results were found while we perform the competing risk regression (SHR, 1.01; 95% CI 0.89–1.12; *P* = 0.947). The primary site of pancreatic tumors was not associated with survival rate, which was demonstrated again by log-rank test and plotted by the Kaplan–Meier survival curve. More details are available from the Fig. 7a, b.

**Table 4:** Patient clinical features before and after propensity score matching

	Before matching			After matching		
Variable	PAH (n = 4877)	PABT (n = 849)	*P* value	PAH (n = 772)	PABT (N = 772)	*P* value
Age, no. (%)			0.293			0.410
18–49	434 (8.9)	87 (10.2)		51 (6.6)	71 (9.2)	
50–59	1215 (24.9)	197 (23.2)		190 (24.6)	176 (22.8)	
60–69	1778 (36.5)	296 (34.9)		288 (37.3)	283 (36.7)	
≥ 70	1450 (29.7)	269 (31.7)		243 (31.5)	242 (31.3)	
Sex, no. (%)			0.022			0.074
Female	2563 (52.6)	410 (48.3)		391 (50.6)	394 (51.0)	
Male	2314 (47.4)	439 (51.7)		381 (49.4)	378 (49.0)	
Race, no. (%)			0.104			0.590
White	4032 (82.7)	679 (80.0)		610 (79.0)	644 (83.4)	
Black	488 (10.0)	104 (12.2)		86 (11.1)	74 (9.6)	
Other	357 (7.3)	67 (7.9)		76 (9.8)	54 (7.0)	
Marital status, no. (%)			0.310			0.283
Never married	603 (12.4)	106 (12.5)		110 (14.2)	90 (11.7)	
Married	3279 (67.2)	589 (69.4)		496 (64.2)	548 (71.0)	
Divorced/widowed	995 (20.4)	154 (18.1)		166 (21.5)	134 (17.4)	
Size, no. (%)			< 0.001			0.866
≤ 2 cm	662 (13.6)	54 (6.4)		45 (5.8)	50 (6.5)	
≤ 4 cm	3135 (64.3)	363 (42.8)		358 (46.4)	354 (45.9)	
> 4 cm	1080 (22.1)	432 (50.9)		369 (47.8)	368 (47.7)	
Differentiation, no. (%)			0.551			0.439
Well	399 (8.2)	78 (9.2)		74 (9.6)	59 (7.6)	
Intermediate	2197 (45.0)	393 (46.3)		351 (45.5)	365 (47.3)	
Poor/undifferentiated	1833 (37.6)	300 (35.3)		294 (38.1)	285 (36.9)	
Missing	448 (9.2)	78 (9.2)		53 (6.9)	63 (8.2)	
Lymph node ratio, no. (%)			0.019			0.889
1–25%	2923 (59.9)	543 (64.0)		505 (65.4)	502 (65.0)	
26–50%	1315 (27.0)	190 (22.4)		167 (21.6)	174 (22.5)	
> 50%	639 (13.1)	116 (13.7)		100 (13.0)	96 (12.4)	
Number of malignant mass, no. (%)			0.305			0.790
One	4684 (96.0)	809 (95.3)		754 (97.7)	744 (96.4)	
More than one	193 (4.0)	40 (4.7)		18 (2.3)	28 (3.6)	

*PAH* pancreatic head tumors, *PABT* pancreatic bodytail tumors

## Discussion

The primary aim of this study was to explore the prevalence of distant metastasis and survival rate for patients with lymph node metastases (LN +) split by primary site. In the population-based cohort derived from the SEER database during a 13 year study, patients with pancreatic head were found to have shorter DM rate compared with pancreatic bodytail, and the survival rate between them was similar. LN + patients with pancreatic head tumors had the 1 year OS 70.6% relative to pancreatic bodytail 1 year OS 69.1%. Multivariable logistic analyses of variables shown that there were significant differences between the groups in the presentation. As for DM + patients with pancreatic head tumor, they were characterized to younger ages and smaller size relative to bodytail counterparts. Multivariable Cox analysis showed that older people, larger size, more advanced differentiation, DM + , higher LNR and more malignant mass were associated with the OS and CSS. At last the PSM confirmed that our conclusion bears the test.

The majority of patients with pancreatic tumors was already in the middle-advanced stage at the time of diagnosis, and one reason is that patients in early stage was lack of early symptoms, another is that the multidetector computed tomography(MDCT) and magnetic resonance imaging (MRI) ability of examination was limited, though they were the most common optimal imaging modality for early pancreatic tumor diagnosis [18]. Therefore, in our study, further research was conducted mainly based on patients who have occurred lymph node metastasis (LN +). Surgical resection was the best treatment to prolong survival time, but for patients with DM + , exploratory laparotomy of pancreatic bring the morbidity up to 42.3% and the survival rate went down, instead of increasing [19]. It is particularly important to determine if the patient has distant metastasis, because the outcome will provide us important clues for individualized prevention and treatment strategies. Furthermore, huge number of patients in middle-advanced stage who have lost the best chance of surgery, other treatments become the first choice, such as chemotherapy [20], so we predict patient prognosis primarily based on clinical information of LN + patients with chemotherapy, which is more in line with the real patient situation. In short, LN + patients with pancreatic cancer are not easy to curable, and a treatment strategy that balances disease control, toxicity, and quality of life is critical.

In our studies, we found the incidence of DM was lower in pancreatic head than in pancreatic bodytail tumors (OR, 0.29; 95% CI 22.8–36.8%; *P* < 0.001), which is consistent to results of univariate analysis after PSM (OR, 0.22; 95% CI 0.15–0.34; *P* < 0.001). Similar results were found from previous studies [5]. However, other studies reported that the risk factor for DM by a multivariate logistic model yielded contradictory results that pancreatic bodytail tumor has a similar incidence of DM compared with pancreatic head tumor [21, 22]. The reason for these contradictory reports may results from inclusion criteria for patients and staging difference. In our study, lymph node metastasis occurred in all of selected patients, as well as chemotherapy. The M1 was defined as tumor distant metastasis according to the TNM classification (American Joint Committee on Cancer, 7th edition) for pancreatic tumors.

The Kaplan–Meier curve with log-rank test was performed to explore the survival rate difference between pancreatic head and pancreatic bodytail tumors without regard to adjustment for other risk factors. We found the pancreatic head has a similar survival rate compared with pancreatic bodytail tumors, while the result was inconsistent with the founding based on the multivariate Cox regression model that pancreatic head tumor has a shorter survival time compared with pancreatic bodytail tumors. It’s fascinating that the primary site wasn’t a risk factor associated with prognosis after competing risk model and PSM. However, previous study show that the survival rate for pancreatic head is higher compared with that pancreatic body tumors in local stage (20 versus 9%), while the survival rate of pancreatic body tumor was higher than pancreatic head in regional (6.7 versus 7.6%) or distant stage (1.4 versus 1.8%) [9]. Interestingly, some studies also found there was no significant association between pancreatic primary site and overall survival based on multivariate Cox regression analyses [23, 24], which is in agreement with our results.

When we delve into other risk factors associated with DM, the results reflect no significant difference in distant metastases in patients under 50. Race also isn’t a risk factor in our founding, and other studies also confirmed this points, but it’s a pity that most of the studies [25, 26] have looked at distant metastases in colorectal cancer. Another noteworthy finding was that for bad differentiated cell type of pancreatic tumors, the pancreatic head tumor didn’t have a difference in rate of DM compared with pancreatic bodytail. Because no relevant studies have been performed to explore the relationship of degree of differentiate with rate of DM, the comparison was difficult to make. Another study [27] confirmed that poorly differentiated cell type has a higher risk of lymph node metastasis in early gastric cancer compared with well/moderately cell type, which shows a yielded contradictory results. The reason for contradictory reports may be result from different types of cancer. When we explore risk factor for DM by primary site, for pancreatic head tumor, women have a significantly higher probability of DM than men in our founding, while a previous study present men have a higher risk of metastasis than women in all pancreatic tumor [5].

When we explore the risk factor for survival rate, black people have a shorter survival time compared with other race, and the results have been demonstrated in many cancers [28–30], but the study found the black people have a better survival outcome when controlling for stage at the time of treatment [29]. Marital status has no difference in patient prognosis for pancreatic tumor, but some studies have reported the variable was a critical factor that could directly affect the clinical prognosis in other cancers [31, 32]. We also explored the risk factors associated with cancer-specific survival, but there is no significant difference between risk factors associated with cancer-specific survival and overall survival. On the other hand, we performed a competing risk model to avoid the death not related to pancreatic tumor, so that we could better compared the prognosis of pancreatic head and pancreatic bodytail tumors. We found age is not related to tumor prognosis, which may tell us that patients with pancreatic tumors in advanced stage have a trend that the majority of patients die of disease not related to pancreatic tumor, but this phenomenon is rare in other tumors [33, 34]. Another discovery worth rethinking is that pancreatic bodytail has a higher risk of DM than pancreatic head tumors for LN + patients, but survival rate didn’t present significant difference. We may allow the following conclusion that the prognosis of LN + patients with pancreatic tumors isn’t associated with the primary site of metastasis, but with the occurrence of metastasis.

This study existed several limitations. Although we used propensity scores matching to compensate for the bias of the retrospective study, there is still the potential for serious selectivity bias when a large number of samples are rejected. Even with statistical adjustment for confounding enrolled variables, unmeasured factors may influence our results in this study. The SEER database provided patients with clinical information which doesn’t include follow-up data on disease recurrence and progression-free survival. In addition, some laboratory tests related to patient prognosis were not included, such CA199, CEA. Lack of these data, we were not able to evaluate the effect of factors on survival rate. Furthermore, we are talking about patients with chemotherapy, but specific chemotherapy drugs are not provided, which will bring the results a great bias in patient prognosis because we know that different chemotherapy drugs have different prognosis effects. However, even with these limitations, we recognize a large number of strengths in our study. When we enrolled patients with chemotherapy, highly statistically significant analysis was derived based on a large sample size available and the results are compelling. Investing the pancreatic head and bodytail may further shed light on disparities in patients care. In any case, there is significant difference in risk factors for DM when split by primary site. Further examination of targeted treatment or care based on primary site are merited. Finally, efforts examining the risk factors may improve the difference of DM rate. Perhaps most important, targeted provision of care in the pancreatic head and bodytail should be a major destination in the future.

## Conclusions

This retrospective study had shown pancreatic head cancer has a lower probability of distant metastasis than pancreatic body cancer, but survival rates didn’t present significant differences between them. This result gives us an important hint that the survival between pancreatic head and pancreatic bodytail tumor for LN + patients with chemotherapy have a similar trend whether or not a DM occurs. In other words, prognosis of LN + patients with chemotherapy isn’t associated with the primary site of metastasis, but with the occurrence of metastasis. Further studies that explore the risk factors of DM and long-term outcomes after different treatment modalities targeting different primary site are warranted.

## Data Availability

The datasets analyzed in this study are obtained from SEER database to extract the eligible cases. The data are also available by contacting the corresponding author.

## Abbreviations

CI: Confidence interval
OS: Overall survival
CSS: Cancer–specific survival
LN: Lymph node
LN +: Lymph node positive
DM: Distant metastasis
DM +: Distant metastasis positive
SEER: Surveillance, epidemiology, and End Results
PSM: Propensity score matching
OR: Odd ratios
HR: Hazard ratios
SHR: Subdistribution hazard ratios
PAH: Pancreatic head tumor
PABT: Pancreatic bodytail tumor

## References

[1] Yachida S, Jones S, Bozic I, Antal T, Leary R, Fu B, Kamiyama M, Hruban RH, Eshleman JR, Nowak MA (2010). Distant metastasis occurs late during the genetic evolution of pancreatic cancer. Nature.

[2] Spanknebel K, Conlon KC (2001). Advances in the surgical management of pancreatic cancer. Cancer J.

[3] Siegel RL, Miller KD, Jemal A (2019). Cancer statistics, 2019. CA Cancer J Clin.

[4] Ansari D, Bauden M, Bergstrom S, Rylance R, Marko-Varga G, Andersson R (2017). Relationship between tumour size and outcome in pancreatic ductal adenocarcinoma. Br J Surg.

[5] Liu X, Fu Y, Chen Q, Wu J, Gao W, Jiang K, Miao Y, Wei J (2018). Predictors of distant metastasis on exploration in patients with potentially resectable pancreatic cancer. BMC Gastroenterol.

[6] Beltran L, Gonzalez-Trejo S, Carmona-Herrera DD, Carrillo JF, Herrera-Goepfert R, Aiello-Crocifoglio V, Gallardo-Rincon D, Melendez-Ponce NA, Ochoa-Carrillo FJ, Onate-Ocana LF (2019). Prognostic factors and differences in survival of right and left colon carcinoma: a STROBE compliant retrospective cohort study. Arch Med Res.

[7] Li Y, Zhao L, Gungor C, Tan F, Zhou Z, Li C, Song X, Wang D, Pei Q, Liu W (2019). The main contributor to the upswing of survival in locally advanced colorectal cancer: an analysis of the SEER database. Therap Adv Gastroenterol.

[8] Doubeni CA, Corley DA, Quinn VP, Jensen CD, Zauber AG, Goodman M, Johnson JR, Mehta SJ, Becerra TA, Zhao WK (2018). Effectiveness of screening colonoscopy in reducing the risk of death from right and left colon cancer: a large community-based study. Gut.

[9] Lau MK, Davila JA, Shaib YH (2010). Incidence and survival of pancreatic head and body and tail cancers: a population-based study in the United States. Pancreas.

[10] Sener SF, Fremgen A, Menck HR, Winchester DP (1999). Pancreatic cancer: a report of treatment and survival trends for 100,313 patients diagnosed from 1985–1995, using the National Cancer Database. J Am Coll Surg.

[11] Birnbaum DJ, Bertucci F, Finetti P, Birnbaum D, Mamessier E (2019). Head and body/tail pancreatic carcinomas are not the same tumors. Cancers (Basel).

[12] Sohn TA, Yeo CJ, Cameron JL, Koniaris L, Kaushal S, Abrams RA, Sauter PK, Coleman J, Hruban RH, Lillemoe KD (2000). Resected adenocarcinoma of the pancreas-616 patients: results, outcomes, and prognostic indicators. J Gastrointest Surg.

[13] Dalton RR, Sarr MG, van Heerden JA, Colby TV (1992). Carcinoma of the body and tail of the pancreas: is curative resection justified?. Surgery.

[14] Tanaka T, Ikeda M, Okusaka T, Ueno H, Morizane C, Hagihara A, Iwasa S, Kojima Y (2008). Prognostic factors in japanese patients with advanced pancreatic cancer treated with single-agent gemcitabine as first-line therapy. Jpn J Clin Oncol.

[15] Marechal R, Demols A, Gay F, De Maertelaere V, Arvanitaki M, Hendlisz A, Van Laethem JL (2007). Prognostic factors and prognostic index for chemonaive and gemcitabine-refractory patients with advanced pancreatic cancer. Oncology.

[16] Warren JL, Klabunde CN, Schrag D, Bach PB, Riley GF (2002). Overview of the SEER-Medicare data: content, research applications, and generalizability to the United States elderly population. Med Care.

[17] Edge SB, Compton CC (2010). The American Joint Committee on cancer the 7th edition of the AJCC Cancer Staging Manual and the future of TNM. Ann Surg Oncol.

[18] Kikuyama M, Kamisawa T, Kuruma S, Chiba K, Kawaguchi S, Terada S, Satoh T (2018). Early diagnosis to improve the poor prognosis of pancreatic cancer. Cancers (Basel).

[19] Insulander J, Sanjeevi S, Haghighi M, Ivanics T, Analatos A, Lundell L, Del Chiaro M, Andren-Sandberg A, Ansorge C (2016). Prognosis following surgical bypass compared with laparotomy alone in unresectable pancreatic adenocarcinoma. Br J Surg.

[20] Neoptolemos JP, Kleeff J, Michl P, Costello E, Greenhalf W, Palmer DH (2018). Therapeutic developments in pancreatic cancer: current and future perspectives. Nat Rev Gastroenterol Hepatol.

[21] Karabicak I, Satoi S, Yanagimoto H, Yamamoto T, Hirooka S, Yamaki S, Kosaka H, Inoue K, Matsui Y, Kon M (2016). Risk factors for latent distant organ metastasis detected by staging laparoscopy in patients with radiologically defined locally advanced pancreatic ductal adenocarcinoma. J Hepatobiliary Pancreat Sci.

[22] Dreyer SB, Jamieson NB, Upstill-Goddard R, Bailey PJ, McKay CJ, Biankin AV, Chang DK (2018). Defining the molecular pathology of pancreatic body and tail adenocarcinoma. Br J Surg.

[23] Maeda S, Ariake K, Iseki M, Ohtsuka H, Mizuma M, Nakagawa K, Morikawa T, Hayashi H, Motoi F, Kamei T (2019). Prognostic indicators in pancreatic cancer patients undergoing total pancreatectomy. Surg Today.

[24] Moutardier V, Magnin V, Turrini O, Viret F, Hennekinne-Mucci S, Goncalves A, Pesenti C, Guiramand J, Lelong B, Giovannini M (2004). Assessment of pathologic response after preoperative chemoradiotherapy and surgery in pancreatic adenocarcinoma. Int J Radiat Oncol Biol Phys.

[25] Zhu C, You Y, Liu S, Ji Y, Yu J (2020). A nomogram to predict distant metastasis for patients with esophageal cancer. Oncol Res Treat.

[26] Gaitanidis A, Alevizakos M, Tsaroucha A, Tsalikidis C, Pitiakoudis M (2018). Predictive nomograms for synchronous distant metastasis in rectal cancer. J Gastrointest Surg.

[27] Choi AH, Nelson RA, Merchant SJ, Kim JY, Chao J, Kim J (2016). Rates of lymph node metastasis and survival in T1a gastric adenocarcinoma in Western populations. Gastrointest Endosc.

[28] Benson KRK, Aggarwal S, Carter JN (2020). Predicting survival for patients with metastatic disease. Int J Radiat Oncol Biol Phys.

[29] Heller DR, Nicolson NG, Ahuja N, Khan S, Kunstman JW (2019). Association of treatment inequity and ancestry with pancreatic ductal adenocarcinoma survival. JAMA Surg.

[30] Bradley CJ, Eguchi M, Perraillon MC (2019). Factors associated with utilization of high cost agents for the treatment of metastatic non-small cell lung cancer. J Natl Cancer Inst.

[31] Sharon CE, Sinnamon AJ, Ming ME, Chu EY, Fraker DL, Karakousis GC (2018). Association of marital status with T stage at presentation and management of early-stage melanoma. JAMA Dermatol.

[32] Bernard B, Burnett C, Sweeney CJ, Rider JR, Sridhar SS (2020). Impact of age at diagnosis of de novo metastatic prostate cancer on survival. Cancer.

[33] Rombouts AJM, Hugen N, Elferink MAG, Poortmans PMP, Nagtegaal ID, de Wilt JHW (2019). Increased risk for second primary rectal cancer after pelvic radiation therapy. Eur J Cancer.

[34] Li ZY, Zhang QW, Teng LM, Zhang CH, Huang Y (2019). Comparable rates of lymph node metastasis and survival between diffuse type and intestinal type early gastric cancer patients: a large population-based study. Gastrointest Endosc.

